# Brain structural associations of syntactic complexity and diversity across schizophrenia spectrum and major depressive disorders, and healthy controls

**DOI:** 10.1038/s41537-024-00517-6

**Published:** 2024-11-01

**Authors:** Katharina Schneider, Nina Alexander, Andreas Jansen, Igor Nenadić, Benjamin Straube, Lea Teutenberg, Florian Thomas-Odenthal, Paula Usemann, Udo Dannlowski, Tilo Kircher, Arne Nagels, Frederike Stein

**Affiliations:** 1 Department of English and Linguistics, General Linguistics, University of Mainz, Mainz, Germany; 2 Department of Psychiatry and Psychotherapy, University of Marburg, Marburg, Germany; 3 Center for Mind, Brain and Behavior, University of Marburg, Marburg, Germany; 4 Institute for Translational Psychiatry, University of Münster, Münster, Germany

**Keywords:** Schizophrenia, Psychiatric disorders

## Abstract

Deviations in syntax production have been well documented in schizophrenia spectrum disorders (SSD). Recently, we have shown evidence for transdiagnostic subtypes of syntactic complexity and diversity. However, there is a lack of studies exploring brain structural correlates of syntax across diagnoses. We assessed syntactic complexity and diversity of oral language production using four Thematic Apperception Test pictures in a sample of *N* = 87 subjects (*n* = 24 major depressive disorder (MDD), *n* = 30 SSD patients both diagnosed according to DSM-IV-TR, and *n* = 33 healthy controls (HC)). General linear models were used to investigate the association of syntax with gray matter volume (GMV), fractional anisotropy (FA), axial (AD), radial (RD), and mean diffusivity (MD). Age, sex, total intracranial volume, group, interaction of group and syntax were covariates of no interest. Syntactic diversity was positively correlated with the GMV of the right medial pre- and postcentral gyri and with the FA of the left superior-longitudinal fasciculus (temporal part). Conversely, the AD of the left cingulum bundle and the forceps minor were negatively correlated with syntactic diversity. The AD of the right inferior-longitudinal fasciculus was positively correlated with syntactic complexity. Negative associations were observed between syntactic complexity and the FA of the left cingulum bundle, the right superior-longitudinal fasciculus, and the AD of the forceps minor and the left uncinate fasciculus. Our study showed brain structural correlates of syntactic complexity and diversity across diagnoses and HC. This contributes to a comprehensive understanding of the interplay between linguistic and neural substrates in syntax production in psychiatric disorders and HC.

## Introduction

Deviant thinking, language, and communication, collectively known as formal thought disorder (FTD), have been extensively documented within schizophrenia spectrum disorders (SSD), encompassing the diagnoses schizophrenia (SZ) and schizoaffective disorder (SZA)^1,2^. Multiple studies highlighted the clinical importance of FTD in SSD as it predicts onset, recurring episodes, and hospitalization^2,3^. Furthermore, FTD has been associated with poor (social)functioning^4^ and perceived quality of life^5^. More recently, this multifaceted construct has been investigated across SSD as well as major depressive disorder (MDD) and bipolar disorder showing transdiagnostic dimensions of FTD (i.e., disorganization, emptiness, and incoherence) and underlying neuroanatomical structures^6,7^. FTD has a prevalence rate of up to 80% in SZ, 60% in SZA, 53% in MDD, and 6% in healthy controls (HC)^3^. While investigations of speech and language disturbances (i.e., FTD) assessed with operational rating scales such as the rating scale for the assessment of objective and subjective formal thought and language disorder (TALD)^8^ are of high clinical value, they do not facilitate the evaluation of nuanced phenomena or deviations concerning language structure including grammar and sound organization. Therefore, studies have used computational linguistic tool such as natural language processing (NLP) and derived latent features for investigating subtle alterations in language production and processing including syntactic and semantic domains^9–14^. While these studies successfully reported several NLP latent features (e.g., semantic coherence^15,16^, T-units, nominal subjects per clause^14^) to be altered in SSD^17^, studies using transdiagnostic approaches in this field remain scarce^7,18^.

Many studies have highlighted large commonalities across different psychiatric disorders. These include the (i) psychopathological overlap: Despite distinct diagnostic criteria, MDD and SSD share several symptomatic features. For example, both disorders present with cognitive impairments, anhedonia, and deficits in social functioning for an example see^19^. (ii) environmental and genetic commonalities: Recent genetic studies have identified shared genetic risk factors between MDD and SSD. For instance, genetic variations associated with both disorders suggest common underlying biological mechanisms^6,20–22^, as well as environmental factors such as childhood maltreatment, pregnancy and perinatal risk factors^23^. Moreover, a recent meta-analysis showed psychotic symptoms, depressive symptoms, anxiety, disruptive behaviors, affective lability, and sleep problems to be transdiagnostic antecedents^24^. (iii) neuroimaging similarities: Both MDD and SSD exhibit reductions in gray matter volume (GMV) in overlapping brain regions, such as the prefrontal cortex and hippocampus^25,26^. Abnormalities in white matter volume and brain dysconnectivity^6,22,27^ were also reported in both disorders, indicating similar structural brain changes^21,28,29^. (iv) language dysfunctions: Language impairments, including difficulties with syntax and semantics, are present in both MDD and SSD patients, further supporting the rationale for a combined analysis^7,30^. Given the symptomatic^19^, genetic^6,20–22^, cognitive^31^, brain structural^6,25^, and environmental risk^23^ commonalities across affective and psychotic disorders, transdiagnostic-dimensional approaches are a key in progressing our understanding of psychiatric disorders. Therefore, our study adopts a dimensional approach aligned with the Research Domain Criteria (RDoC)^32^ framework as well as Hierarchical Taxonomy of Psychopathology (HiTOP)^33,34^, which both aim to investigate the underlying neurobiological and behavioral dimensions across traditional diagnostic boundaries. By focusing on shared features and underlying mechanisms rather than categorical diagnoses, we aim to uncover more fundamental insights into the relevance of speech and language across psychiatric disorders^19,21,32,33,35^.

While semantic coherence, sentence length and use of determiner^15,16^ have been reported to be predictive for transition to psychosis^36^, specifically impairments in syntactic complexity have been linked to diminished social cognition^37^ and impaired social functioning^38^ in SSD. Syntactic complexity refers to the use of subordinate clauses. At the most basic syntactic level, a sentence can exist as a main clause without any embedded segments, while complex sentences are formed by combining multiple clauses^39^. These complex sentences can be viewed as a fusion of coordinated structures, wherein distinct parts of sentences are linked by conjunctions, and subordinate structures, which entail a main clause accompanied by at least one subordinate clause that relies on the main clause for its meaning. The utilization of subordinate clauses empowers speakers to effectively and coherently communicate information^40^. Consequently, a decrease in language complexity results in a limited ability to articulate thoughts during social interactions^41^, which is associated with disappointment^42^ and less affiliation^43^. Furthermore, incoherent speech might be more likely to trigger negative feelings in the listener^43^. This can result in feeling less close to them or reject them. In contrast, coherent narratives lead to more social interactions and support, which may have a positive impact on psychological well-being^43,44^. Syntactic diversity which is related to but different from syntactic complexity, refers to the different types of subordinate structures such as temporal, local, modal or causal embedded adverbial clauses^45^. Thus, syntactic diversity represents aspects of the syntax-semantics interface due to embedding of subordinate clauses is a syntactic process, whereas selecting the specific type of subordinate clause describes a semantic process^46^. All subordinate clauses provide additional information to the main clause, e.g., a cause, a condition, or a specification^47^. In a previous study of ours, we have investigated measures of syntactic complexity and diversity across HC, MDD, and SSD. In SSD, both syntactic complexity and diversity were notably reduced compared to HC and MDD. Moreover, four different subtypes of syntactic language production were delineated across diagnosis, ranging from extremely complex to slightly complex language while being accompanied by higher and lower FTD as well as cognitive impairment^18^.

Brain structural correlates of language disturbances have been mainly investigated using clinical ratings of FTD scales in SSD. Hereof, the GMV of the bilateral superior temporal gyri, the middle temporal gyri, and inferior frontal gyri have been consistently reported to be negatively associated with FTD symptoms^1,48,49^. In prior studies, various white matter fiber tracts have been linked to FTD, including but not limited to the inferior longitudinal fasciculus (ILF), arcuate fasciculus (AF), the left uncinate fasciculus (UF), the superior longitudinal fasciculus (SLF), the inferior fronto-occipital fasciculus (IFOF), the cingulum bundle (CB), and the anterior thalamic radiation^50,51^. Associations between FTD and brain structure are not unique to SSD. Recently, we have identified transdiagnostic correlates of FTD dimensions in patients with MDD, SSD, and bipolar disorder investigating GMV, white matter microstructure, and structural connectomics^6,25^. The present study builds on this transdiagnostic approach by investigating syntactic complexity and diversity derived from a spontaneous language production task across diagnoses. De Boer and colleagues investigated the association of syntactic measures and white matter microstructure showing the bilateral IFOF and AF to be implicated in syntactic processing^17^ in SSD. However, (i) this study only included SSD patients and HC; (ii) used a broader concept for assessing alterations of syntactic language production, that comprises all sentence structures, not only complex sentences; and (iii) analyzed exclusively the integrity of white matter tracts.

Investigating language production features such as syntactic complexity and diversity as well as their association to brain structure will enable researchers to identify crucial links between linguistic deficits and neuroanatomical alterations in psychiatric disorders. Identifying these structural associations can help pinpoint specific neural pathways and regions implicated in language production and processing, thus fostering a more comprehensive understanding of the intricate interplay between cognitive, linguistic, and neural mechanisms underlying affective and psychotic disorders. Therefore, we investigate brain structural correlates of syntactic complexity and diversity in spontaneous language production in MDD, SSD and HC using voxel-based morphometry (VBM) and tract-based spatial statistics (TBSS)^52^ approaches. We will expand previous studies by (i) applying a cross-diagnostic approach and (ii) using an in-depth characterization of alterations in syntax (i.e., complexity and diversity) in association with gray and white matter brain structure. Using whole-brain-analysis, we hypothesize associations between syntax and brain structure in regions being part of the language network and previously reported in the context of FTD. Based on previous transdiagnostic studies, we expect these associations to be present across groups. However, the severity of alterations may differ across MDD, SSD and HC.

## Results

### Association of gray matter volume and syntactic complexity and diversity

**Fig. 1:**
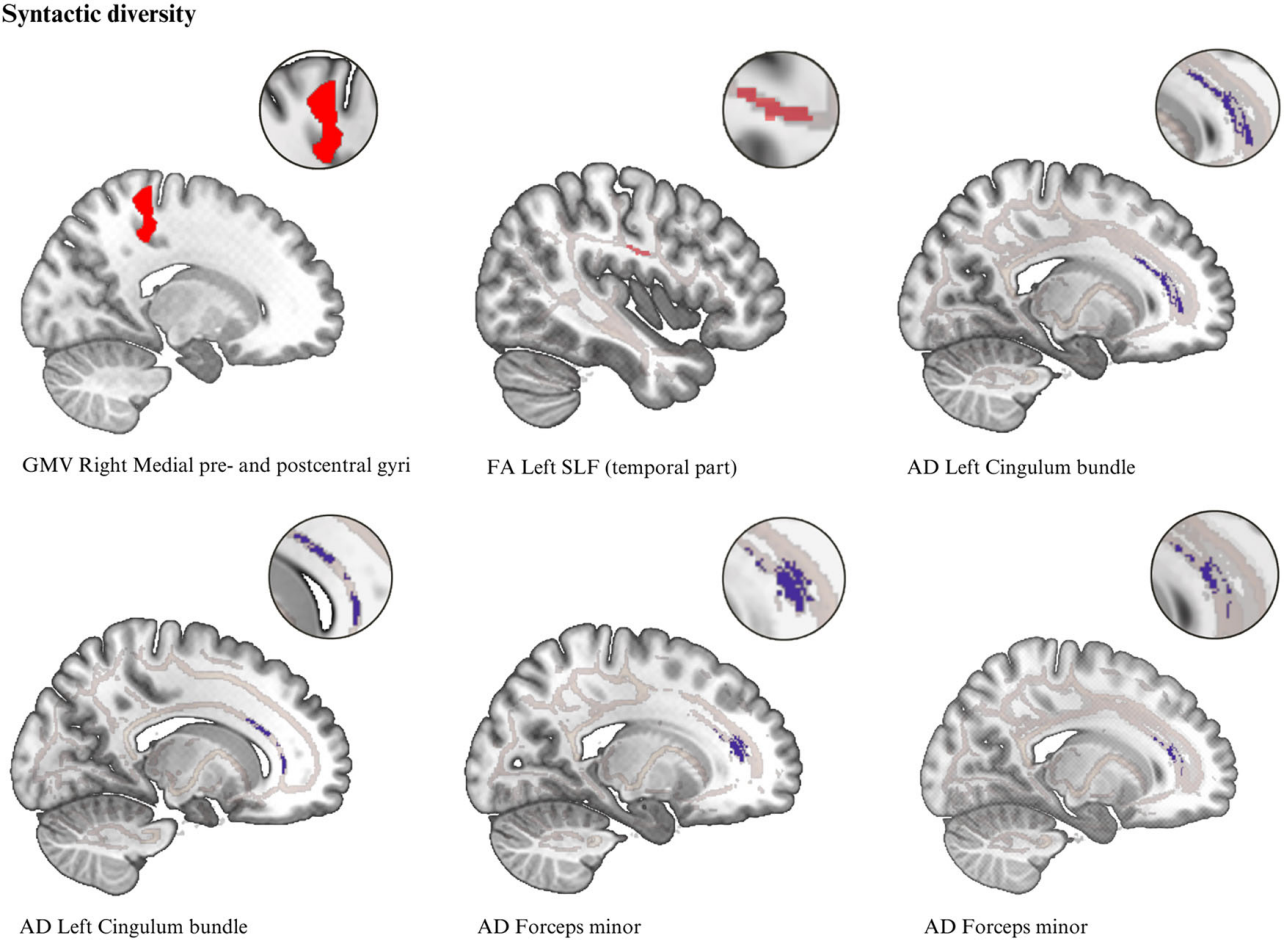
Association of gray matter volume, white matter fractional anisotropy, and axial diffusivity with syntactic diversity. GMV clusters are shown at p_FWE_ < 0.05 cluster-level, DTI clusters are shown at p_TFCE_ < 0.05, family-wise-error corrected.

Syntactic diversity correlated positively with GMV of the right medial pre- and postcentral gyri (*k* = 939, x/y/z = 14/-33/56, *t* = 4.87, *p*_*FWE*_ = 0.025, β = 0.254) (Fig. 1 and Table 1). The cluster of the right medial pre- and postcentral gyri was moderated by years of education and executive functioning (Trail Making Test (TMT^53^)) (*p*s ≤ 0.001). No significant moderation effects of verbal IQ, lateralization as derived by the EHI questionnaire and its lateralization quotient^54^, verbal fluency (VF^55^), number of prompts, duration of current episode, lifetime number and duration of hospitalizations, and medication were detected after correcting for multiple testing (Benjamini & Hochberg) (see Supplementary Table 1). No association between syntactic complexity and GMV was found.

**Table 1:** Associations of syntactic complexity and diversity with gray matter volume and white matter microstructure.

Measure	Correlation	Coordinates of the maximum intensity voxel (x/y/z) MNI	Anatomic labeling	Hemisphere	k	P_FWE_	P adj	β
**Gray matter volume**								
Syntactic diversity	Positive	14/−33/56	Medial pre- and postcentral gyri	Right	939	0.025	**0.041**	0.254
**White matter microstructure**								
**Fractional anisotropy**								
Syntactic diversity	Positive	−46/−10/24	Superior-longitudinal fasciculus (temporal part)	Left	104	0.036	**0.044**	0.311
Syntactic complexity	Negative	−17/36/14	Cingulum bundle	Left	313	0.018	**0.040**	–0.168
	Negative	37/−50/30	Superior-longitudinal fasciculus	Right	38	0.038	**0.044**	–0.141
**Axial diffusivity**								
Syntactic diversity	Negative	−18/41/6	Cingulum bundle	Left	214	0.014	**0.040**	–0.146
	Negative	−16/26/21	Cingulum bundle	Left	130	0.018	**0.040**	
	Negative	−16/37/3	Forceps minor		18	0.040	**0.044**	–0.070
	Negative	−18/41/6	Forceps minor		262	0.018	**0.040**	
Syntactic complexity	Negative	−18/34/19	Forceps minor		332	0.016	**0.040**	–0.258
	Positive	33/−44/14	Inferior-longitudinal fasciculus	Right	19	0.048	**0.048**	0.220
	Negative	−22/38/−4	Uncinate fasciculus	Left	72	0.026	**0.041**	–0.271

Bold font indicates significant results after correcting for multiple testing (Benjamini & Hochberg).

### Association of white matter microstructure and syntactic complexity and diversity

**Fig. 2:**
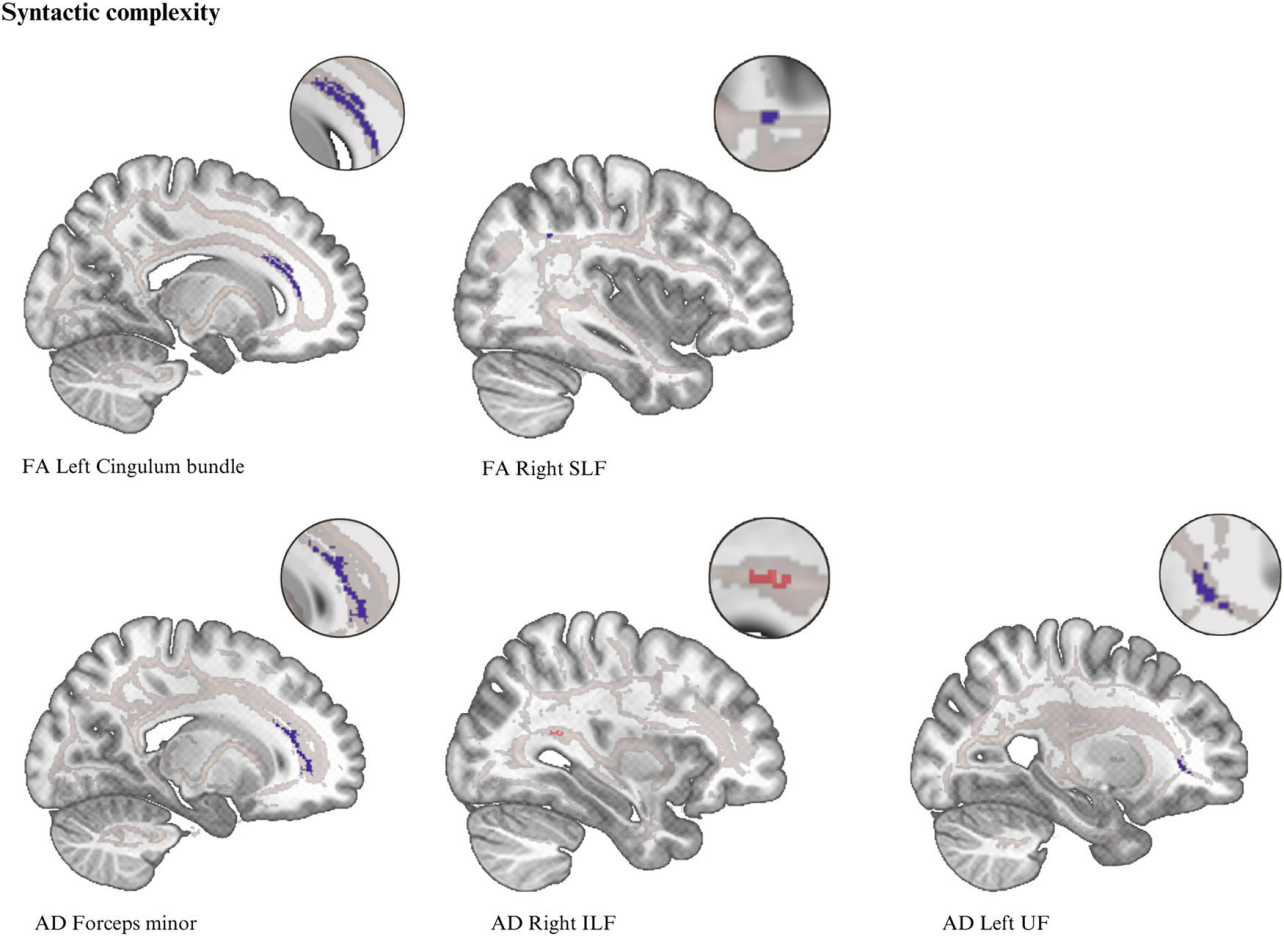
Association of white matter fractional anisotropy and axial diffusivity with syntactic complexity. Clusters are shown at p_TFCE_ < 0.05, family-wise-error corrected.

The association of syntactic complexity and diversity with microstructure white matter fractional anisotropy (FA), axial (AD), radial (RD), and mean diffusivity (MD) was tested using a TBSS^52^ approach. Results of FA and AD are presented in Table 1 and Figs. 1, 2. The correlation of FA left SLF (temporal part) and syntactic diversity was moderated by years of education (*p* = 0.001). No significant moderation effects of verbal IQ, lateralization quotient^54^, TMT^53^, VF^55^, number of prompts, duration of current episode, lifetime number and duration of hospitalizations, and medication were found after correcting for multiple testing (Benjamini & Hochberg) (see Supplementary Table 1). No significant associations were found for RD and MD.

## Discussion

This study investigated the association of syntactic measures with gray and white matter brain structure across MDD, SSD, and HC. We expanded upon the categorical perspective by examining associations of syntax and brain structure dimensionally across different psychiatric conditions and HC while controlling for group, and the interaction of group and syntax. This approach allows the investigation of cross-diagnostic effects, independent of categorical diagnoses. Syntactic diversity was positively correlated with the GMV of the right medial pre- and postcentral gyri and with the FA of the left temporal part of the SLF. Conversely, the AD of the left CB and forceps minor were negatively correlated with syntactic diversity. The AD of the right ILF was positively correlated with syntactic complexity. Negative associations were observed between syntactic complexity and the FA of the left CB, the right SLF, and the AD of the forceps minor and the left UF. Notably, all of these findings were not moderated by verbal IQ, lateralization quotient, verbal fluency, prompts during language production, duration of current episode, lifetime number and duration of hospitalizations, and medication. Moderation effects were available for years of education (GMV right medial pre- and postcentral gyri and FA left SLF) and executive functioning (GMV right medial pre- and postcentral gyri).

Several new insights can be derived from the present study. First, our findings present compelling evidence for cross-diagnostic associations between gray and white brain matter structures in left and right hemispheres with syntactic complexity and diversity observed in oral language production. Associations were present even when controlling for group (MDD, SSD, and HC) demonstrating diagnoses-shared neural correlates with both linguistic measures. This finding aligns with previous research showing transdiagnostic correlates of FTD as the behavioral measures of speech abnormalities^6,19,25,56^ as well as research on general overlaps across mental disorders^23,24^.

Second, in addition to distinct syntactic neuroanatomical correlates, i.e., diversity was correlated with the right medial pre- and postcentral gyri GMV, syntactic complexity was associated with the AD of the right ILF and left UF, our results show brain structures associated with both syntactic measures, i.e., the FA of the SLF, the AD of the forceps minor, and the FA and AD left CB were associated with syntactic complexity and diversity. This might indicate a broader syntactic information transfer of these white matter tracts.

Third, our study found that the right pre- and postcentral gyri GMV were positively related to syntactic diversity, which aligns with previous research linking these brain regions to various language abilities. For instance, one study identified the right pre- and postcentral gyri as part of a bilateral network activated during the production of more complex structures, highlighting their role in managing linguistic complexity^57^. A systematic review reported an association between right pre- and postcentral gyri GMV and negative FTD in SZ^49^. Furthermore, a structural covariance network analysis demonstrated a correlation between the caudate nucleus and the right postcentral gyrus with naming abilities and executive functioning in older adults, indicating their broader cognitive significance^58^. Interestingly, this association was positively moderated by years of education and negatively by executive functioning in our analyses, indicating an increase of the positive correlation between syntactic diversity and right pre- and postcentral gyri GMV with higher level of education, while higher executive functioning decreased this association. The moderation by years of education is also supported by a study showing syntactic abilities to be increased with high level of education^59^. Strong executive functioning, especially in domains such as planning and inhibition, can improve the ability to handle complex tasks, including syntactic production^60^. However, if individuals become excessively focused on performing tasks that require high cognitive effort, they may consider it challenging to achieve the fluidity and creativity necessary for syntactic diversity. This indicates that, although executive functions are advantageous, an excessive emphasis on control and structure may impede spontaneous syntactic expression. Finally, the right pre- and postcentral gyri are integral to both the motor^61^ and cognitive aspects^62^ of language, supporting the production of complex and diverse syntactic structures. Their involvement in broader cognitive functions, such as executive functioning, further underscores their importance in facilitating syntactic diversity in language use^63^.

Fourth, exclusively associated with syntactic complexity were the AD of right ILF and the left UF. The right ILF has been linked to FTD (i.e., disorganization) in a transdiagnostic sample^25^ and plays a significant role in language complexity by supporting the integration of visual and lexical information with connecting temporal and occipital regions^64–66^. Its involvement in these language functions highlights its importance in the broader network of brain regions supporting language processing^65^. The contribution of the UF in relation to language-related functions continues to be controversially discussed^67^. The UF is considered to be part of the brain’s ventral pathways that are predominantly linked to semantic processing in language^68^, but as shown by our study and others, the UF provides the connection between anterior temporal lobe and prefrontal regions and therefore plays an important role in many linguistic areas, e.g., verbal fluency, reading, listening comprehension^69^, and syntactic processes^70,71^. Moreover, its association with social-emotional functioning has been demonstrated for different groups^72–76^. Based on these evidences, we would hypothesize a connection of socio-emotional functioning and syntactic complexity regarding one’s ability to communicate and its social impact^43,44^.

Fifth, the FA of the SLF was associated with both syntactic measures. However, the FA of the left temporal part of the SLF was positively correlated with diversity and the FA of the right SLF was negatively correlated with complexity. The left SLF, which connects frontal and temporal language areas, is crucial for integrating different linguistic elements^77^. Its integrity supports the ability to produce varied and complex syntactic forms, reflecting syntactic diversity^78^. Like GMV, this association was positively moderated by years of education, a finding further supported by a study demonstrating that higher levels of education are linked to enhanced syntactic abilities^59^. The negative correlation between the FA of the right SLF and syntactic complexity indicates that higher FA in this region is associated with less syntactic complexity. This might suggest that the right SLF, typically less involved in syntactic production compared to the left hemisphere^69^, could play a compensatory or inhibitory role in managing syntactic complexity. Alternatively, it could reflect a lateralization effect where the right SLF’s increased integrity might be linked to other cognitive functions that do not prioritize syntactic complexity. Additionally, the FA and AD of the left CB and the forceps minor were negatively associated with syntactic complexity and diversity. The CB has been associated with cognitive functions including working memory, attention, emotional prosody, problem-solving and executive processes^79–82^. These cognitive functions are essential for handling complex syntactic structures, as they support the integration and manipulation of linguistic information. Some associations of the CB overlap with those of the forceps minor which is involved in emotion experience, syntactic performance, understanding of written metaphors, and has been associated with auditory hallucinations in SZ^71,80,83,84^. The negative correlations with syntactic complexity and diversity emphasize the role of these tracts in facilitating the cognitive and emotional processes in connected brain regions required for complex language tasks. Exploring these relationships yields valuable insights into the neural mechanisms that underpin language production.

Finally, this study included five different brain structural metrics encompassing voxel-based analyses of GMV and four white matter microstructure measures (i.e., FA, AD, MD, RD). This multifaceted approach allowed for a comprehensive investigation into the brain metrics implicated in syntactic language production. We did not find any correlations between syntax and MD as well as RD, but we were able to detect multiple associations with FA or AD across different fiber tracts. Furthermore, the FA and AD of the left CB were negatively correlated with syntax. Both diffusion tensor imaging (DTI) metrics provide insights into white matter microstructure; however, FA primarily indicates the integrity of fiber tracts, while AD measures the diffusivity of water molecules along the axis of these tracts^85,86^. Although FA and AD may occasionally produce overlapping results due to common structural characteristics and pathological changes, they can also differ significantly because of their unique sensitivities to white matter microstructure and the complexity of fiber architecture^87^. AD is the more sensitive measure of axonal integrity, enabling a greater number of associations to be identified for AD compared to FA^87^. In sum, our results contribute to the understanding of the neural underpinnings of syntax and highlight the importance of considering multiple brain structural metrics to capture the complexity of brain-behavior relationships. Future research should further explore these associations in larger and more diverse samples.

### Limitations

Several limitations should be acknowledged. First, our sample size was relatively small, and the range of age was quite broad. Second, while some studies have reported the influence of psychiatric medication on syntax, others have not^14,18,88^. We did not find any moderation between the detected GMV, FA, and AD clusters with psychiatric medication. Nevertheless, we only included acute medication and potential effects related to the lifetime intake of psychiatric medication can therefore not be ruled out. Third, this study employed a cross-sectional, correlational design, which precludes making causal inferences. Fourth, using manual analysis for assessing syntactic complexity and diversity, as opposed to employing NLP algorithms, has certain drawbacks, including reduced comparability and efficiency. Nevertheless, the depth of analysis required made a manual approach the most suitable choice. Fifth, while our study excluded individuals with severe somatic conditions, such as cancer, autoimmune diseases, and neurological disorders, it is important to acknowledge that the potential effects of underlying or undiagnosed somatic diseases on our results cannot be entirely excluded. Sixth, other syntactic measures could have yielded different results.

## Conclusion

This study provides significant insights into the neural correlates of syntactic complexity and diversity across different groups, including MDD, SSD, and HC. Our findings reveal compelling evidence of cross-diagnostic associations between gray and white matter structures in both hemispheres, highlighting shared neural substrates underlying syntactic abilities. Notably, distinct brain structural correlates were identified for syntactic diversity (i.e., right medial pre- and postcentral gyri GMV) and complexity (i.e., right ILF and left UF). Furthermore, the study identified brain structures linked to both syntactic measures (i.e., SLF, forceps minor, and left CB). These findings contribute to a deeper understanding of the interplay between brain structure and syntactic language production, emphasizing the need for further research to explore these relationships across various populations and contexts.

## Methods

### Participants

For the present study, we included *N* = 87 (HC = 33, MDD = 24, SSD = 30) German-speaking participants (aged 20–65) who were part of the FOR2107 MACS cohort. The SSD group subdivides into particular diagnoses, i.e., paranoid SZ (*n* = 8), disorganized SZ (*n* = 2), undifferentiated SZ (*n* = 2), residual SZ (*n* = 2), SZA (*n* = 8), depressive type of SZA (*n* = 3), and bipolar type of SZA (*n* = 5). None of the MDD patients had psychotic features as assessed according to DSM-IV-TR. Participants were enrolled from both inpatient and outpatient units at the Marburg University Hospital, as well as from departments at nearby local hospitals within a 50 km range. Additionally, recruitment was extended through announcements in local newspapers and distribution of flyers. The study applied specific exclusion criteria, including verbal IQ < 80, a history of head trauma or unconsciousness, severe medical conditions such as cancer, autoimmune diseases, infections, neurological disorders (e.g., stroke, dementia), ongoing substance dependence, and current intake of benzodiazepines. The local Ethics Committee granted approval for all protocols in accordance with the principles outlined in the Declaration of Helsinki. Before joining the study, patients provided written informed consent and were also provided with financial compensation. A summary of descriptive statistics is presented in Table 2.

**Table 2:** Descriptive statistics.

	HC (*n* = 33)	MDD (*n* = 24)	SSD (*n* = 30)	Comparison (*p)*	P adj	Effect size (*η²)*
Age	42.03 (13.74)	40.46 (11.54)	41.43 (13.44)	0.904	0.964	0.002
Sex	f = 20 m = 13	f = 14 m = 10	f = 10 m = 20	0.065	0.087	
Syntactic complexity	0.40 (0.12)	0.42 (0.13)	0.33 (0.09)	0.004^a^	**0.007**	0.121
Syntactic diversity	0.63 (0.12)	0.64 (0.14)	0.54 (0.14)	0.008^a^	**0.013**	0.110
Total no. of words	1131.48 (319.74)	1176.33 (367.70)	1062.80 (440.78)	0.538	0.615	0.015
Total no. of sentences	64.73 (17.57)	64.67 (20.54)	79.53 (35.98)	0.047	0.068	0.070
Years of education	15.38 (2.61)	13.17 (2.57)	11.78 (2.14)	<0.001^b^	**0.002**	0.290
SANS sum score	0.20 (0.58)	5.74 (7.68)	17.07 (11.64)	<0.001^c^	**0.002**	0.451
SANS alogia	0.07 (0.37)	0.83 (1.23)	2.23 (2.51)	<0.001^c^	**0.002**	0.245
SAPS sum score	0.21 (0.83)	1.23 (2.07)	13.24 (13.40)	<0.001^c^	**0.002**	0.362
SAPS pFTD	0.07 (0.37)	1.48 (2.35)	6.43 (8.24)	<0.001^c^	**0.002**	0.253
HAM-D sum score	0.86 (1.75)	4.65 (6.31)	7.96 (6.14)	<0.001^d^	**0.002**	0.284
YMRS sum score	0.21 (0.69)	0.62 (1.20)	5.93 (6.61)	<0.001^c^	**0.002**	0.267
GAF score	90.83 (9.75)	67.50 (12.90)	53.54 (14.49)	<0.001^e^	**0.002**	0.699
Verbal IQ	117.96 (13.57)	112.13 (13.46)	110.55 (14.43)	0.116	0.143	0.055
TIV	1561.67 (157.48)	1540.69 (174.41)	1541.29 (126.81)	0.970	0.970	0.001

Means and standard deviations (SD) (in brackets) are listed for each group and category.

Bold font indicates significant results after correcting for multiple testing (Benjamini & Hochberg).

*SANS* Scale for Assessment of Negative Symptoms, *SAPS* Scale for Assessment of Positive Symptoms, *HAM-D* Hamilton Rating Scale for Depression, *YMRS* Young Mania Rating Scale, *GAF* Global Assessment of Functioning, *TIV* Total Intracranial Volume.

One-way ANOVA and Tukey’s HSD (post hoc test) were used for pairwise comparisons:

^a^SSD < MDD, HC.

^b^SSD, MDD < HC.

^c^SSD > MDD, HC.

^d^SSD > HC.

^e^SSD < MDD < HC.

### Data assessment

Clinical diagnosis (according to DSM-IV-TR) as well as psychopathological scales were assessed as part of a semi-structured interview. DSM-IV-TR instead of the newer DSM-V was used due to (i) data acquisition for this study started in 2014 and (ii) comparability with previous cohort studies running at the Department. Clinical information including duration of current episode, lifetime number and duration of hospitalizations, age of onset and duration of illness were assessed as part of the clinical interview and upon medical records if available (see Supplementary Table 2).

All interviewers were well-versed in and underwent training to proficiently evaluate clinical diagnosis and psychopathological scales. Interrater reliability was evaluated using the interclass coefficient, demonstrating strong reliability with values exceeding r > 0.86 for all scales used. For MDD and SSD patients, we tested associations with the intake of psychiatric medication using the medication load index. We used the medication load index as reported by previous studies^89–91^. The advantage of this score is the inclusion of type of medication, i.e., antidepressants plus antipsychotic amongst others (i.e., any psychotropic medication) as well as the dosages. In summary, this score is computed by coding the dose of each psychotropic medication (e.g., antidepressant, mood stabilizer, antipsychotic and anxiolytic medication) as absent = 0, low = 1 or high = 2. Low- or high-dose groupings were performed using a previously employed approach^92^.

Syntactic complexity and diversity were assessed as described in Schneider et al.^18^. In summary, we employed four Thematic Apperception Test (TAT) pictures (picture 1, 2, 4, and 6), following the procedures outlined by Liddle et al.^93^. Instead of utilizing eight one-minute spontaneous speech samples, we conducted assessments with four distinct TAT pictures, each lasting three minutes. This approach aimed to capture additional speech-related abbreviations that might not have been evident within the initial one-minute window but could emerge over a longer time span of free speech production. Participants were asked to tell a narrative based on each picture, separated by one-minute breaks. The instruction was reiterated before presenting the subsequent picture. In instances where participants concluded their story before the three-minute timeframe, non-directive prompts (e.g., “How do people feel?”; “What could happen next?”) were used by the instructor. Audio recordings of the speech samples were transcribed manually, verbatim using the f4transkript software (https://www.audiotranskription.de/f4transkript/) while being unaware of clinical diagnoses. Next, transcripts were analyzed for linguistic features, including total number of sentences and 13 different types of complex sentences (main clause in combination with subordinate clause) by K.S. Sentence boundaries were set as strictly as possible based on the syntactic structure. If the syntactic information was not clear, then prosodic information was used. If there was still a vague sentence boundary, semantic context was considered. The procedure of sentence segmentation was identical for all transcripts of participants. These types of complex sentences were included in the analysis: temporal, local, modal, causal, conditional, adversative, final, consecutive, concessive, relative, complement, comparative clauses, and indirect questions^18^ (see Supplementary Table 3). As described in previous studies^18,45^, we extracted the relative sum of subordinate clauses as a measure reflecting syntactic complexity, i.e., the ratio of main clauses containing an arbitrary number of subordinate clauses to the total number of sentences. Accordingly, the differentiation of complex sentences from simple sentences was focused, but various levels of embedding were disregarded. Furthermore, the number of different types of subordinate sentences (e.g., causal, conditional, complement clauses) in relation to the total number of analyzed types (i.e., 13) was used as a measure of syntactic diversity.

Structural MRI data were obtained using a 3 T MRI scanner from Siemens, Erlangen, Germany (Tim Trio model) and a 12-channel head matrix Rx-coil. Both acquisition and processing of MRI data followed a comprehensive quality assurance protocol. All assessments were performed within seven days to exclude any time effects. T1-weighted images were acquired using a fast gradient-echo MP-RAGE sequence with a slice thickness of 1.0 mm consisting of 176 sagittal orientated slices and a field of view (FOV) of 256 mm. Scanning parameters were set as follows: time of acquisition (AT): 4.26 min, time of repetition (TR): 1.9 s, time of echo (TE): 2.26 ms, inversion time: 900 ms, flip angle: 9°. DTI scans were assessed using an epi2d sequence with a final voxel resolution of 2.5 × 2.5 × 2.5 mm3. For all participants, two DTI data sets with a different phase encoding direction were acquired, i.e., one set for Anterior-to-Posterior (AP) and one set for Posterior-to-Anterior (PA) phase-encoding. Each set included 30 diffusion-weighted images with b-value 1000 s/mm2 and four non-diffusion-weighted images with b-value 0 s/mm2 (AT: 4.10 min., TR: 7300 ms, TE: 90 ms, FOV: 320 mm, Echo spacing 0.75 ms, 56 slices with 3 mm, flip angle = 60°).

### MRI data preprocessing

For all preprocessing procedures of T1-weighted images, default parameters provided by CAT12 (Computational Anatomy Toolbox for SPM, build 1184, developed by Christian Gaser and the Structural Brain Mapping Group at Jena University Hospital, Germany, available at http://dbm.neuro.uni-jena.de/cat/) were employed. These included the application of a spatial adaptive non-local means (SANLM) denoising filter, followed by internal resampling to harmonize low-resolution images with varying spatial resolutions. Subsequent to correcting intensity inhomogeneities caused by magnetic field variations (also referred to as bias correction^94^) in T1-weighted images, the images were segmented into white matter (WM), gray matter (GM), and cerebrospinal fluid (CBF) using the standard unified segmentation method^95^. In a refined voxel-based processing phase, further steps were conducted. These included skull-stripping and regional parcellation of the brain into left and right hemispheres, cerebellum, and subcortical areas based on the segmented data. Additionally, potential local white matter hyperintensities were identified. Subsequently, a local intensity transformation was applied to all tissue classes, followed by a final maximum a posteriori estimation of tissue segmentation^96^. To calculate the proportion of each tissue type within each voxel, a partial volume estimation technique was utilized^97^. Lastly, spatial normalization was carried out using the DARTEL method^98^. This involved registering all images to a common template by estimating a 12-parameter affine transformation. Images were smoothed with a Gaussian kernel of 8 mm FWHM.

Prior to preprocessing, a visual examination was conducted on all DTI scans to identify significant artifacts or caliber gaps. Hereof, 13 participants (HC = 4, MDD = 3, SSD = 6) had to be excluded for DTI analyses. A TBSS^52^ approach was employed, facilitated by FSL (version 6.0; the Oxford Center for Functional Magnetic Imaging Software Library; Oxford, UK). Data underwent preprocessing utilizing default parameters. During this phase, corrections were applied to address motion-related issues and Eddy-Current artifacts. The images were subsequently non-linearly registered to the standard Montreal Neurological Institute (MNI) space using an appropriate FSL template. Finally, FA, AD, RD, and MD maps were projected onto average skeletons, with a 0.2 threshold set to prevent misalignments.

### Statistical analyses

To investigate the association of syntactic complexity and diversity with GMV and FA, AD, RD, MD, we used separate general linear models per modality and syntactic measure. All models included age, sex, total intracranial volume, group, interaction of group and syntax, as covariates of no interest plus syntactic diversity and complexity, respectively.

VBM analyses were conducted utilizing SPM12 (v6906), running under Matlab (2019b). Consistent with recommended VBM procedures, we applied an absolute threshold masking using a threshold value of 0.1, as advised by the CAT12 toolbox’s guidelines (http://dbm.neuro.uni-jena.de/cat/). Statistical significance of results was set at a cluster-level family-wise-error-corrected (FWE) threshold of *p* < 0.05 to account for multiple comparisons. This threshold was applied subsequent to an initial threshold of *p* < 0.001 uncorrected. The assignment of anatomical labels to clusters was accomplished using the Dartel space Neuromorphometrics and AAL atlases.

Tract-based analysis of DTI data was performed using the threshold-free cluster enhancement (TFCE) method. To generate the GLM contrasts, a total of 5000 permutations were executed, following established protocols (https://fsl.fmrib.ox.ac.uk). The Johns Hopkins University (JHU) ICBM-DTI-81 white-matter labels atlas and the JHU white-matter tractography atlas^99,100^ were employed for precise cluster labeling. MNI coordinates were ascertained using the FSL cluster tool. Statistical significance was set at a threshold of *p* < 0.05, corrected for family-wise error (FWE). For both GMV and DTI, Benjamini & Hochberg^101^ correction was applied for multiple testing.

Eigenvariates as approximated means of significant GMV and FA, AD clusters were extracted for post hoc moderation analyses. Moderation analyses (Jamovi (v2.3)) were used to investigate the potential moderating effects of years of education, verbal IQ (assessed with the Mehrfachwahl-Wortschatz-Intelligenztest version B (MWT-B)^102^), lateralization, cognitive performance (i.e., executive functioning assessed with the TMT^53^ and VF test (semantic, phonemic, and alternating)^55^), duration of current episode, lifetime number and duration of hospitalizations, and medication load index on the detected brain structural correlates. Analyses were corrected for multiple testing using the Benjamini & Hochberg^101^ approach.

## Supplementary information

Supplementary Information

## Data Availability

The data and code supporting the findings of this study can be accessed by contacting the corresponding author (K.S.).

## Code availability

The data and code supporting the findings of this study can be accessed by contacting the corresponding author (K.S.).
