# Author Correction: Brain structural associations of syntactic complexity and diversity across schizophrenia spectrum and major depressive disorders, and healthy controls

**DOI:** 10.1038/s41537-024-00530-9

**Published:** 2024-11-18

**Authors:** Katharina Schneider, Nina Alexander, Andreas Jansen, Igor Nenadić, Benjamin Straube, Lea Teutenberg, Florian Thomas-Odenthal, Paula Usemann, Udo Dannlowski, Tilo Kircher, Arne Nagels, Frederike Stein

**Affiliations:** 1 Department of English and Linguistics, General Linguistics, University of Mainz, Mainz, Germany; 2 Department of Psychiatry and Psychotherapy, University of Marburg, Marburg, Germany; 3 Center for Mind, Brain and Behavior, University of Marburg, Marburg, Germany; 4 Institute for Translational Psychiatry, University of Münster, Münster, Germany

**Keywords:** Schizophrenia, Psychiatric disorders

Correction to: *Schizophrenia* 10.1038/s41537-024-00517-6, published online 1 November 2024

In this article, the last sentence of the Acknowledgements section, should have read ‘This study is funded by the Research Units of General Linguistics and Language Typology at the University of Mainz.’ The original article has been corrected.

