# Supplementary material for: Brain structural associations of syntactic complexity and diversity across schizophrenia spectrum and major depressive disorders, and healthy controls
Source: Schizophrenia (Heidelb). 2024 Nov 1;10(1):101. doi: 10.1038/s41537-024-00517-6 (PMC11530549; doi:10.1038/s41537-024-00517-6)
Supplement: Supplementary file 1 — Supplementary Information [file 41537_2024_517_MOESM1_ESM.docx]

**Brain structural associations of syntactic complexity and diversity across schizophrenia spectrum and major depressive disorders, and healthy controls**

**SUPPLEMENTARY INFORMATION**

**TABLES**

**Supplementary Table 1.** Moderation estimates of brain structure, syntax, years of education, verbal IQ, lateralization, cognitive performance, number of prompts, duration of current episode, number of hospitalizations, duration of hospitalization, and medication load index

|  | **Estimate** | **SE** | **Z** | **p** | **P adj** |
| --- | --- | --- | --- | --- | --- |
|  |  |  |  |  |  |
| **Years of education** |  |  |  |  |  |
| GMV Right Medial pre- and postcentral gyri:  Syntactic diversity | .07224 | .01675 | 4.314 | <.001 | **.005** |
| FA Left Superior-longitudinal fasciculus (temporal part): Syntactic diversity | .04822 | .01478 | 3.26 | .001 | **.005** |
| FA Left Cingulum bundle:  Syntactic complexity | .00477 | .02029 | .235 | .814 | .814 |
| FA Right Superior-longitudinal fasciculus:  Syntactic complexity | -.03525 | .02170 | -1.624 | .104 | .234 |
| AD Left Cingulum bundle:  Syntactic diversity | -3.423e-5 | 2.311e-5 | -1.481 | .143 | .257 |
| AD Forceps minor:  Syntactic diversity | -2.114e-5 | 2.201e-5 | -.961 | .340 | .383 |
| AD Forceps minor:  Syntactic complexity | -2.872e-5 | 2.296e-5 | -1.251 | .215 | .323 |
| AD Right Inferior-longitudinal fasciculus:  Syntactic complexity | 7.942e-5 | 3.704e-5 | 2.139 | .036 | .108 |
| AD Left Uncinate fasciculus:  Syntactic complexity | -2.843e-5 | 2.678e-5 | -1.062 | .292 | .376 |
| **Verbal IQ** |  |  |  |  |  |
| GMV Right Medial pre- and postcentral gyri:  Syntactic diversity | -9.52e−4 | .00334 | -.285 | .776 | .776 |
| FA Left Superior-longitudinal fasciculus (temporal part): Syntactic diversity | .00223 | .00299 | .746 | .456 | .693 |
| FA Left Cingulum bundle:  Syntactic complexity | -.00217 | .00504 | -.430 | .667 | .750 |
| FA Right Superior-longitudinal fasciculus:  Syntactic complexity | -.00486 | .00561 | -.867 | .386 | .693 |
| AD Left Cingulum bundle:  Syntactic diversity | 4.911e-6 | 4.343e-6 | 1.131 | .262 | .693 |
| AD Forceps minor:  Syntactic diversity | 2.547e-6 | 4.127e-6 | .617 | .539 | .693 |
| AD Forceps minor:  Syntactic complexity | 6.750e-6 | 5.979e-6 | -1.129 | .263 | .693 |
| AD Right Inferior-longitudinal fasciculus:  Syntactic complexity | 1.865e-6 | 9.967e-6 | 1.872 | .066 | .594 |
| AD Left Uncinate fasciculus:  Syntactic complexity | -4.881e-6 | 7.302e-6 | -.668 | .506 | .693 |
| **Lateralization** |  |  |  |  |  |
| GMV Right Medial pre- and postcentral gyri:  Syntactic diversity | .000 | .001 | .218 | .827 | .905 |
| FA Left Superior-longitudinal fasciculus (temporal part): Syntactic diversity | 5.54e-4 | 6.81e-4 | .814 | .416 | .905 |
| FA Left Cingulum bundle:  Syntactic complexity | -4.08e−4 | .00154 | -.266 | .791 | .905 |
| FA Right Superior-longitudinal fasciculus:  Syntactic complexity | -.00216 | .00167 | -1.2911 | .197 | .591 |
| AD Left Cingulum bundle:  Syntactic diversity | -1.890e-6 | 1.041e-6 | -1.816 | .074 | .562 |
| AD Forceps minor:  Syntactic diversity | -1.537e-6 | 9.892e-7 | -1.554 | .125 | .562 |
| AD Forceps minor:  Syntactic complexity | 5.372e-7 | 1.791e-6 | .300 | .765 | .905 |
| AD Right Inferior-longitudinal fasciculus:  Syntactic complexity | -1.753e-6 | 2.840e-6 | -.617 | .539 | .905 |
| AD Left Uncinate fasciculus:  Syntactic complexity | 2.476e-7 | 2.069e-6 | .120 | .905 | .905 |
| **Executive functioning (TMT)** |  |  |  |  |  |
| GMV Right Medial pre- and postcentral gyri:  Syntactic diversity | -.00609 | .00179 | -3.40 | <.001 | **.009** |
| FA Left Superior-longitudinal fasciculus (temporal part): Syntactic diversity | -.00297 | .00157 | -1.895 | .058 | .174 |
| FA Left Cingulum bundle:  Syntactic complexity | .00315 | .00239 | 1.32 | .188 | .423 |
| FA Right Superior-longitudinal fasciculus:  Syntactic complexity | -3.37e−6 | .00273 | -.00124 | .999 | .999 |
| AD Left Cingulum bundle:  Syntactic diversity | 9.906e-7 | 2.832e-6 | .321 | .749 | .963 |
| AD Forceps minor:  Syntactic diversity | 3.455e-7 | 2.675e-6 | .129 | .898 | .999 |
| AD Forceps minor:  Syntactic complexity | 3.190e-6 | 3.792e-6 | .841 | .403 | .605 |
| AD Right Inferior-longitudinal fasciculus:  Syntactic complexity | 1.307e-5 | 6.182e-6 | -2.113 | .038 | .171 |
| AD Left Uncinate fasciculus:  Syntactic complexity | -4.381e-6 | 4.580e-6 | -.957 | .342 | .605 |
| **Semantic VF** |  |  |  |  |  |
| GMV Right Medial pre- and postcentral gyri:  Syntactic diversity | .01045 | .00733 | 1.426 | .154 | .672 |
| FA Left Superior-longitudinal fasciculus (temporal part): Syntactic diversity | .00806 | .00662 | 1.22 | .224 | .672 |
| FA Left Cingulum bundle:  Syntactic complexity | -.00138 | .00836 | -.165 | .869 | .869 |
| FA Right Superior-longitudinal fasciculus:  Syntactic complexity | -.00637 | .00856 | -.744 | .457 | .823 |
| AD Left Cingulum bundle:  Syntactic diversity | -8.703e-6 | 9.968e-6 | -.873 | .386 | .823 |
| AD Forceps minor:  Syntactic diversity | -3.835e-6 | 9.387e-6 | -.409 | .684 | .869 |
| AD Forceps minor:  Syntactic complexity | -1.720e-5 | 1.003e-5 | -1.714 | .091 | .672 |
| AD Right Inferior-longitudinal fasciculus:  Syntactic complexity | 5.491e-6 | 1.716e-5 | .320 | .750 | .869 |
| AD Left Uncinate fasciculus:  Syntactic complexity | -2.053e-6 | 1.209e-5 | -.170 | .866 | .869 |
| **Phonemic VF** |  |  |  |  |  |
| GMV Right Medial pre- and postcentral gyri:  Syntactic diversity | .00853 | .01087 | .785 | .433 | .762 |
| FA Left Superior-longitudinal fasciculus (temporal part): Syntactic diversity | .00466 | .00948 | .492 | .623 | .762 |
| FA Left Cingulum bundle:  Syntactic complexity | -.00330 | .01415 | -.233 | .816 | .816 |
| FA Right Superior-longitudinal fasciculus:  Syntactic complexity | -.00642 | .01542 | -.4162 | .677 | .762 |
| AD Left Cingulum bundle:  Syntactic diversity | -1.320e-5 | 1.369e-5 | -.964 | .339 | .762 |
| AD Forceps minor:  Syntactic diversity | -6.786e-6 | 1.295e-5 | -.524 | .602 | .762 |
| AD Forceps minor:  Syntactic complexity | -2.847e-5 | 1.663e-5 | -1.712 | .092 | .495 |
| AD Right Inferior-longitudinal fasciculus:  Syntactic complexity | 4.454e-5 | 2.748e-5 | 1.621 | .110 | .495 |
| AD Left Uncinate fasciculus:  Syntactic complexity | -9.374e-6 | 2.079e-5 | -.451 | .654 | .762 |
| **Alternating VF** |  |  |  |  |  |
| GMV Right Medial pre- and postcentral gyri:  Syntactic diversity | .02764 | .01108 | 2.493 | .013 | .059 |
| FA Left Superior-longitudinal fasciculus (temporal part): Syntactic diversity | .02490 | .00937 | 2.66 | .008 | .059 |
| FA Left Cingulum bundle:  Syntactic complexity | -.00365 | .01493 | -.245 | .807 | .807 |
| FA Right Superior-longitudinal fasciculus:  Syntactic complexity | -.00745 | .01549 | -.481 | .631 | .743 |
| AD Left Cingulum bundle:  Syntactic diversity | -1.699e-5 | 1.514e-5 | -1.122 | .266 | .479 |
| AD Forceps minor:  Syntactic diversity | -8.764e-6 | 1.419e-5 | -.618 | .539 | .743 |
| AD Forceps minor:  Syntactic complexity | -2.038e-5 | 1.805e-5 | -1.129 | .263 | .479 |
| AD Right Inferior-longitudinal fasciculus:  Syntactic complexity | 4.998e-5 | 3.078e-5 | 1.624 | .109 | .327 |
| AD Left Uncinate fasciculus:  Syntactic complexity | 9.891e-6 | 2.243e-5 | .441 | .661 | .743 |
| **Number of prompts** |  |  |  |  |  |
| GMV Right Medial pre- and postcentral gyri:  Syntactic diversity | -.001 | .018 | -.066 | .947 | .964 |
| FA Left Superior-longitudinal fasciculus (temporal part): Syntactic diversity | -.027 | .015 | -1.748 | .081 | .729 |
| FA Left Cingulum bundle:  Syntactic complexity | -.009 | .021 | -.439 | .661 | .964 |
| FA Right Superior-longitudinal fasciculus:  Syntactic complexity | .020 | .023 | .871 | .384 | .964 |
| AD Left Cingulum bundle:  Syntactic diversity | -1.070e-6 | 2.376e-6 | -.045 | .964 | .964 |
| AD Forceps minor:  Syntactic diversity | -6.116e-6 | 2.251e-5 | -.272 | .787 | .964 |
| AD Forceps minor:  Syntactic complexity | 3.002e-5 | 2.425e-5 | 1.238 | .220 | .964 |
| AD Right Inferior-longitudinal fasciculus:  Syntactic complexity | -2.836e-5 | 4.117e-5 | -.689 | .493 | .964 |
| AD Left Uncinate fasciculus:  Syntactic complexity | 7.008e-6 | 2.913e-5 | .241 | .811 | .964 |
| **Duration of current episode^1^** |  |  |  |  |  |
| GMV Right Medial pre- and postcentral gyri:  Syntactic diversity | .00333 | .00150 | 2.216 | .027 | .243 |
| FA Left Superior-longitudinal fasciculus (temporal part): Syntactic diversity | 6.02e-4 | .00183 | .3285 | .743 | .955 |
| FA Left Cingulum bundle:  Syntactic complexity | .00102 | .00290 | .352 | .725 | .955 |
| FA Right Superior-longitudinal fasciculus:  Syntactic complexity | 1.65e-4 | .00328 | .0502 | .960 | .960 |
| AD Left Cingulum bundle:  Syntactic diversity | 1.683e-6 | 3.807e-6 | .442 | .663 | .955 |
| AD Forceps minor:  Syntactic diversity | 1.418e-6 | 3.591e-6 | .395 | .697 | .955 |
| AD Forceps minor:  Syntactic complexity | 2.804e-6 | 5.646e-6 | .497 | .625 | .955 |
| AD Right Inferior-longitudinal fasciculus:  Syntactic complexity | 1.304e-6 | 7.463e-6 | .175 | .863 | .960 |
| AD Left Uncinate fasciculus:  Syntactic complexity | 6.278e-6 | 5.857e-6 | 1.072 | .296 | .955 |
| **Number of hospitalizations^1^** |  |  |  |  |  |
| GMV Right Medial pre- and postcentral gyri:  Syntactic diversity | -.00964 | .01298 | -.743 | .458 | .589 |
| FA Left Superior-longitudinal fasciculus (temporal part): Syntactic diversity | -.00903 | .01131 | -.799 | .424 | .589 |
| FA Left Cingulum bundle:  Syntactic complexity | .04886 | .03485 | 1.402 | .161 | .492 |
| FA Right Superior-longitudinal fasciculus:  Syntactic complexity | -.00743 | .03748 | -.198 | .843 | .843 |
| AD Left Cingulum bundle:  Syntactic diversity | 2.909e-5 | 1.989e-5 | 1.462 | .151 | .492 |
| AD Forceps minor:  Syntactic diversity | 2.690e-5 | 1.898e-5 | 1.417 | .164 | .492 |
| AD Forceps minor:  Syntactic complexity | 3.144e-5 | 4.904e-5 | .641 | .525 | .591 |
| AD Right Inferior-longitudinal fasciculus:  Syntactic complexity | -7.026e-5 | 6.768e-5 | -1.038 | .305 | .589 |
| AD Left Uncinate fasciculus:  Syntactic complexity | -4.786e-5 | 5.228e-5 | -.915 | .365 | .589 |
| **Duration of hospitalization^1^** |  |  |  |  |  |
| GMV Right Medial pre- and postcentral gyri:  Syntactic diversity | -.00322 | .00269 | -1.20 | .231 | .680 |
| FA Left Superior-longitudinal fasciculus (temporal part): Syntactic diversity | -.00262 | .00246 | -1.063 | .288 | .680 |
| FA Left Cingulum bundle:  Syntactic complexity | .00324 | .00413 | .784 | .433 | .680 |
| FA Right Superior-longitudinal fasciculus:  Syntactic complexity | -.00332 | .00443 | -.750 | .453 | .680 |
| AD Left Cingulum bundle:  Syntactic diversity | 2.034e-6 | 5.082e-6 | .400 | .691 | .888 |
| AD Forceps minor:  Syntactic diversity | 2.501e-7 | 4.805e-6 | .052 | .959 | .959 |
| AD Forceps minor:  Syntactic complexity | 1.315e-6 | 6.418e-6 | .205 | .839 | .944 |
| AD Right Inferior-longitudinal fasciculus:  Syntactic complexity | -9.104e-6 | 9.017e-6 | -1.010 | .319 | .680 |
| AD Left Uncinate fasciculus:  Syntactic complexity | -8.350e-6 | 6.969e-6 | -1.198 | .238 | .680 |
| **Medication load index^1^** |  |  |  |  |  |
| GMV Right Medial pre- and postcentral gyri:  Syntactic diversity | .022 | .042 | .530 | .596 | .621 |
| FA Left Superior-longitudinal fasciculus (temporal part): Syntactic diversity | .04857 | .03730 | 1.302 | .193 | .248 |
| FA Left Cingulum bundle:  Syntactic complexity | .08412 | .05868 | 1.433 | .152 | .228 |
| FA Right Superior-longitudinal fasciculus:  Syntactic complexity | .09134 | .06084 | 1.50 | .133 | .228 |
| AD Left Cingulum bundle:  Syntactic diversity | 1.377e-4 | 6.352e-5 | 2.169 | .036 | .108 |
| AD Forceps minor:  Syntactic diversity | 1.543e-4 | 5.924e-5 | 2.605 | .013 | .090 |
| AD Forceps minor:  Syntactic complexity | 1.948e-4 | 8.072e-5 | 2.414 | .020 | .090 |
| AD Right Inferior-longitudinal fasciculus:  Syntactic complexity | -5.834e-5 | 1.171e-4 | -.498 | .621 | .621 |
| AD Left Uncinate fasciculus:  Syntactic complexity | 1.804e-4 | 8.931e-5 | 2.020 | .050 | .113 |

TMT = Trail Making Test; VF = Verbal fluency

^1^ Moderation analyses using duration of current episode, number of hospitalizations, duration of hospitalization, and medication load index as moderators was carried out for patients (MDD and SSD) exclusively.

Bold font indicates significant results after correcting for multiple testing (Benjamini & Hochberg).

**Supplementary Table 2.** Clinical information of MDD and SSD

|  | **MDD**  **(*n*=24)** | **SSD**  **(*n*=30)** | **Comparison (*p)*** | **P adj** | **Effect size (*ɛ^2^*)** |
| --- | --- | --- | --- | --- | --- |
| Duration of current episode^1^  (in months) | 13.67 (19.2) | 41.1 (80.2) | .695 | .695 | .006 |
| Duration of hospitalization  (in months) | 6.88 (10.3) | 23.8 (26.5) | .003 | **.007** | .170 |
| Number of hospitalizations (lifetime) | 1.46 (2.5) | 3.3 (3.11) | .004 | **.007** | .155 |
| Age of onset  (in years) | 28.45 (15.16) | 17.28 (7.99) | .009 | **.011** | .141 |
| Duration of Illness  (in years) | 14.1 (11.42) | 23.66 (12.44) | .003 | **.007** | .189 |

Means and standard deviations (SD) (in brackets) are listed for each group and category. Due to assumptions for parametric testing were not given, non-parametric Kruskal-Wallis test was used.

Bold font indicates significant results after correcting for multiple testing (Benjamini & Hochberg).

^1^ A current episode refers to the patient’s present state of illness, characterized by a cluster of symptoms.

**Supplementary Table 3.** Examples for all 13 types of complex sentences in German and English

|  | **German** | | **English** |
| --- | --- | --- | --- |
| Temporal clause | Nachdem das Kind aufgestanden ist, hat es sich die Geige genommen. | After the boy got up, he took the violin. | |
| Local clause | Der Vogel bleibt, wo er ist. | The bird stays where it is. | |
| Modal clause | Die Frau spart Geld, indem sie jeden Monat die Hälfte ihres Lohns weglegt. | The woman saves money by putting away half of her salary each month. | |
| Causal clause | Der Mann ist traurig, weil er verlassen wurde. | The man is sad because he has been abandoned. | |
| Conditional clause | Wenn sie wieder gesund ist, dann geht sie morgen einkaufen. | When she's well again, she'll go shopping tomorrow. | |
| Adversative clause | Ich muss laufen, während die anderen Kinder mit dem Bus fahren. | I have to walk while the other children take the bus. | |
| Final clause | Er geht zur Ärztin, damit er eine Diagnose bekommt. | He goes to the doctor to get a diagnosis. | |
| Consecutive clause | Das Feuer ist so heiß, sodass ich ohne Pullover daneben sitzen kann. | The fire is so hot that I can sit next to it without a sweater. | |
| Concessive clause | Obwohl es schon hell, bleibe ich im Bett liegen. | Although it is already light, I stay in bed. | |
| Relative clause | Der Hund, der an der Leine läuft, bellt ganz laut. | The dog on the lead barks loudly. | |
| Complement clause | Sie sagte, dass sie die Nachricht gelesen hat. | She said that she had read the message. | |
| Comparative clause | Das Fahrrad sieht aus, als ob es lange nicht geputzt wurde. | The bike looks as if it hasn't been cleaned for a long time. | |
| Indirect question | Der Gärtner fragt sich, ob er dieses Jahr wieder so viele Tomaten ernten kann. | The gardener wonders whether he will be able to harvest so many tomatoes again this year. | |
